# Alcohol septal ablation for hypertrophic obstructive cardiomyopathy: do mitral valve leaflet length, septal thickness, or sex affect the outcome?

**DOI:** 10.1007/s12928-024-01014-4

**Published:** 2024-05-28

**Authors:** Mesud Mustafic, Rebecka Jandér, David Marlevi, Anette Rickenlund, Andreas Rück, Nawzad Saleh, Sam Abdi, Maria J. Eriksson, Anna Damlin

**Affiliations:** 1 Division of Clinical Physiology, Department of Molecular Medicine and Surgery, Karolinska Institutet, Stockholm, Sweden; 2 Department of Clinical Physiology, Karolinska University Hospital, Karolinska University Hospital, Stockholm, Sweden; 3 Department of Cardiology, Karolinska University Hospital, Karolinska University Hospital, Stockholm, Sweden; 4 Unit of Cardiology, Department of Medicine Solna, Karolinska Institutet, Stockholm, Sweden; 5 Department of Internal Medicine, Karolinska University Hospital, Stockholm, Sweden; 6 Institute for Medical Engineering and Science, Massachusetts Institute of Technology, Cambridge, MA 02139 USA

**Keywords:** Alcohol septal ablation, Echocardiography, Hypertrophic obstructive cardiomyopathy, Basal septal wall thickness, Anterior mitral valve leaflet, Sex differences

## Abstract

This retrospective cohort study aimed to assess whether basal septal wall thickness (BSWT), anterior (AML) and posterior (PML) mitral leaflet length, or sex were associated with remaining left ventricular outflow tract obstruction (LVOTO) in patients with hypertrophic obstructive cardiomyopathy (HOCM) undergoing alcohol septal ablation (ASA). One hundred fifty-four patients who underwent ASA at the Karolinska University Hospital in Stockholm, Sweden, between 2009 and 2021, were included retrospectively. Anatomical and hemodynamic parameters were collected from invasive catheterization before and during ASA, and from echocardiography (ECHO) examinations before, during, and at 1-year follow-up after ASA. Linear and logistic regression models were used to assess the association between sex, BSWT, AML, PML, and outcome, which was defined as the remaining LVOTO (≥ 30 mmHg) after ASA. The median follow-up was 364 days (interquartile range 334–385 days). BSWT ≥ 23 mm (*n* = 13, 12%) was associated with remaining LVOTO at follow-up (*p* = 0.004). Elongated mitral valve leaflet length (either AML or PML) was present in 125 (90%) patients. Elongated AML (> 24 mm) was present in 67 (44%) patients, although AML length was not associated with remaining LVOTO at follow-up. Elongated PML (> 14 mm) was present in 114 (74%) patients and was not associated with remaining LVOTO at follow-up. No significant sex differences were observed regarding the remaining LVOTO. ECHO measurement of BSWT can be effectively used to select patients for successful ASA and identify those patients with a risk of incomplete resolution of LVOTO after ASA.

**Graphical abstract:**

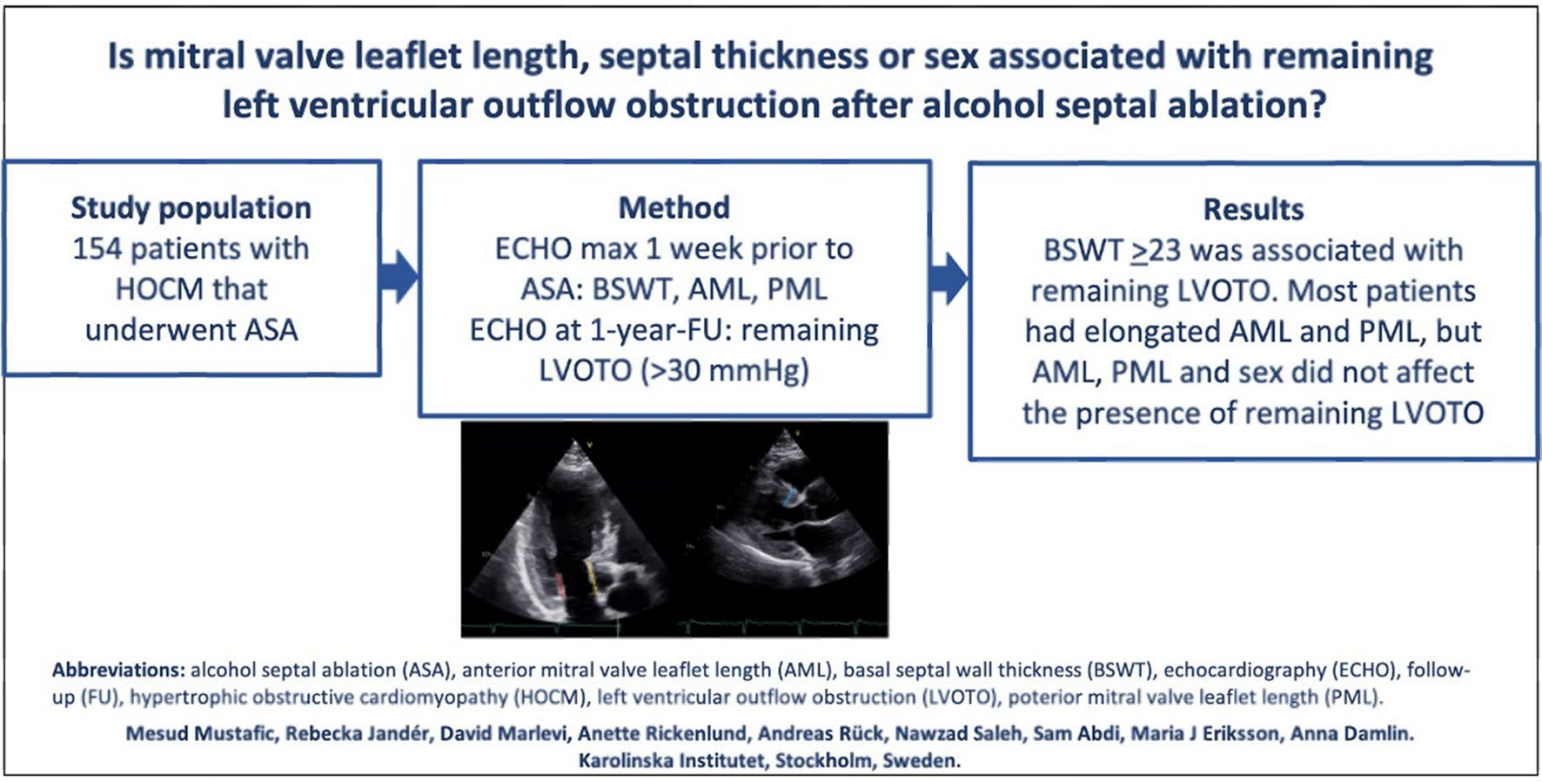

## Introduction

The most severe form of hypertrophic cardiomyopathy (HCM) is hypertrophic obstructive cardiomyopathy (HOCM), where basal septal hypertrophy contributes to left ventricular outflow tract (LVOT) obstruction (LVOTO). In addition, patients with HOCM are also at increased risk of arrhythmias [1]. In addition to the septum hypertrophy, systolic anterior motion (SAM) and mitral valve leaflet (MVL) abnormalities are the other known pathophysiological mechanisms underlying LVOTO in patients with HOCM [2, 3]. All the available treatment options for HOCM are designed to reduce LVOTO-associated symptoms [1]. However, traditional medical therapies using beta-blockers, calcium antagonists, and/or disopyramide are not disease specific. The recently introduced selective cardiac myosin inhibitor, mavacamten, decreases the myocardial hypercontractility present in patients with HOCM [1]. However, this inhibitor is not yet clinically approved in Sweden. Compared with placebo treatment, patients with HOCM who receive mavacamten have shown improved exercise capacity and health status, as well as decreases in LVOTO and New York Health Association (NYHA) functional class [1]. As a result, the European Society of Cardiology now recommends that mavacamten should be considered as a second-line therapy for HOCM, when optimal traditional medical therapy is ineffective or poorly tolerated [1]. Where pharmacological treatment cannot mitigate patients’ severe symptoms and disease progress, septal reduction therapies, such as surgical myectomy or alcohol septal ablation (ASA), are the recommended interventions for the treatment of these patients’ HOCM and LVOTO. Ventricular septal myectomy is the most commonly performed surgical procedure to treat HOCM, where a rectangular trough is created in the basal septum, including the point of mitral leaflet-septal contact, to reduce LVOTO [1]. In ASA, a regional percutaneous alcohol injection is performed to induce a focal myocardial infarction in a selected part of the basal left ventricular septal wall, which reduces septal wall thickness and LVOTO [4]. Compared to surgical myectomy, ASA is associated with shorter recovery time, faster intervention time, and fewer risks associated with cardiac surgery [5]. However, the proportion of patients who do not respond to ASA and require further reintervention is higher among patients undergoing ASA (5–25%) compared with those undergoing surgical myectomy (< 1%) [6, 7]. The reason for this higher reintervention rate is not completely understood.

In addition to their hypertrophic septum, patients with HCM may have elongated MVLs compared with healthy control subjects, which leads to a further risk of developing LVOTO [3]. However, the impact of pre-procedural basal septal wall thickness (BSWT) and mitral valve leaflet length (MVLL) on the success rate among patients with HOCM undergoing ASA has been less studied. Furthermore, the literature describing sex differences in ASA results is still limited. Hence, this study aims to investigate if BSWT, MVLL, and sex are associated with remaining LVOTO in patients with HOCM undergoing ASA.

## Methods

### Study design and population

This retrospective cohort study included all 158 patients with HOCM who underwent ASA on following clinical diagnosis at the Karolinska University Hospital in Stockholm, Sweden, between June 2, 2009, and July 1, 2021. A multi-disciplinary HCM team, including clinical and interventional cardiologists, clinical physiologists, and thoracic surgeons discussed the cases of patients who underwent ASA. The decision to proceed with ASA was based on clinical symptoms (NYHA functional class) and significant LVOT gradient as documented by rest or exercise transthoracic Doppler echocardiography (ECHO), despite optimal medical therapies including beta-blockers, calcium antagonists, and/or disopyramide, and in agreement with patient preference. Patients accepted for ASA were considered not suitable for surgical myectomy due to the high perioperative or postoperative risk because of their late age, high comorbidity, significant renal insufficiency and/or morbid obesity, or patient preference. All patients had undergone a pre-procedural ECHO performed at a maximum of 1 week before ASA. One to three follow-up visits with ECHO were performed during the first year after ablation, with additional follow-up if clinically motivated. In this study, if the 1-year follow-up ECHO was not available, the latest study from the interval of 3–24 months post-intervention was included. Patients who were planned for an ASA but who could not undergo the procedure due to their lack of a suitable septal arterial branch were excluded (*n* = 4). Hence, 154 patients were included in the final analysis.

### Data collection

#### Patient data

Patient data and ASA-specific data were collected from patient records and imaging systems, including left-heart catheterization (CATH) (picture archiving and communication system/radiology information system, PACS/RIS), and ECHO data (Viewpoint; GE HealthCare, Chicago, IL, USA). Each patient’s data were collected at three different time points: the pre-procedural ECHO performed at a maximum of 1 week before ASA; CATH and ECHO during the ASA; and a follow-up ECHO 1 year after ASA. Values missing from the patients’ files were registered as missing values.

#### ECHO

All ECHO examinations were performed by personnel who were highly experienced in cardiac imaging using advanced ultrasound machines. Image acquisition was performed according to a standard protocol, including additional images focusing on Doppler and HCM-specific morphological findings (as described below) to enable comprehensive HCM evaluations [8].

#### Pre-procedural ECHO

**Fig. 1:**
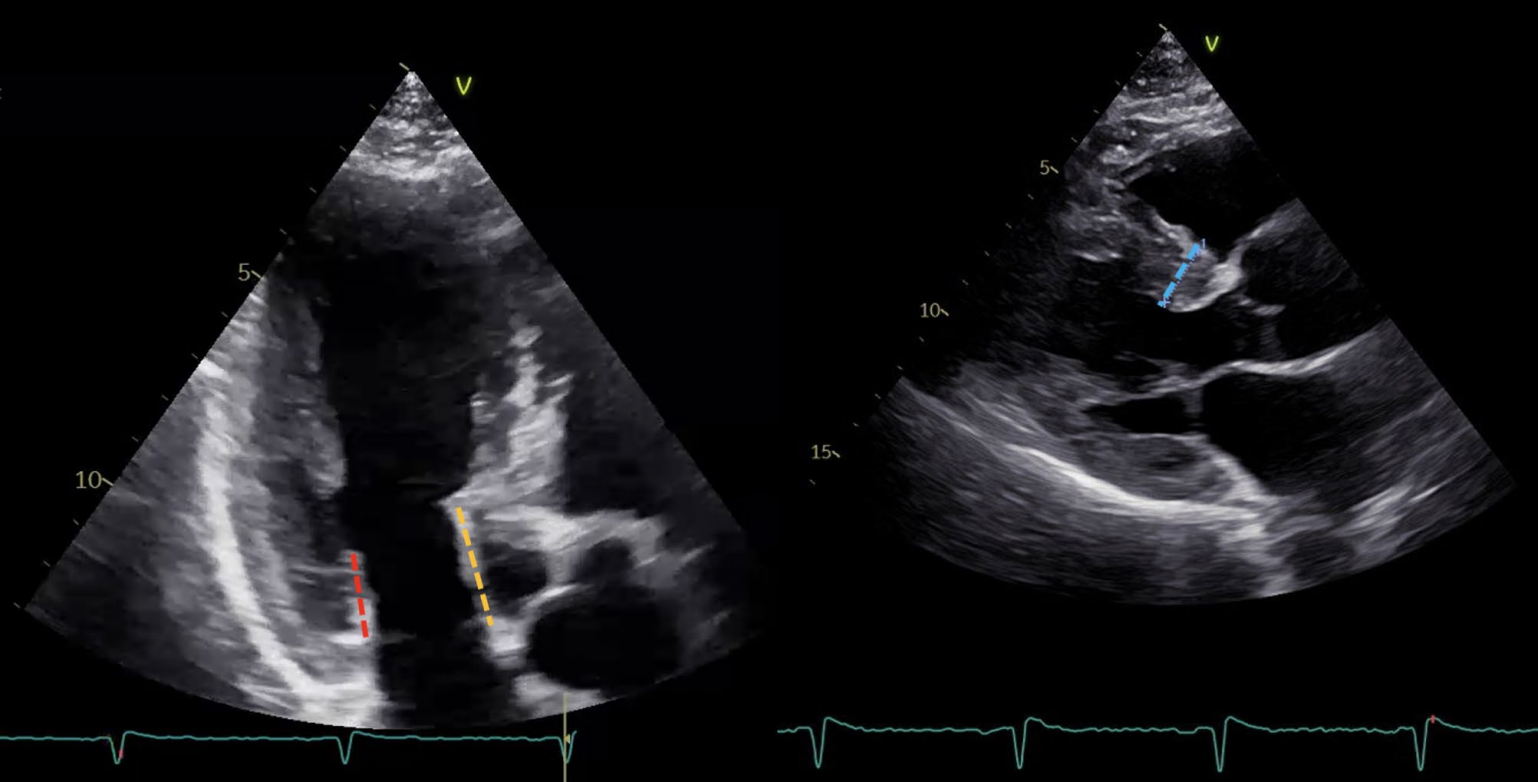
Measures of mitral valve leaflet length and basal septal wall thickness in pre-procedural ECHO images. **A** Mitral valve leaflet length (red line: posterior mitral leaflet length; yellow line: anterior mitral leaflet length). **B** Basal septal wall thickness (blue line)

The pre-ECHO data included: age, sex, heart rate (beats per minute, BPM), weight, length, body surface area (BSA), left ventricular (LV) end-diastolic and end-systolic diameters, LV end-diastolic and end-systolic volume, left atrium (LA) end-systolic volume, LV BSWT (Fig. 1), and posterior wall thickness [9]. Furthermore, the mitral and aortic regurgitation grade, presence of SAM, LVOT peak systolic velocity, and gradients at rest and Valsalva, estimated from LVOT velocity using a simplified Bernoulli equation (Δ*P* = 4*V*^2^) were collected. For the MV-specific assessment, the preoperative ECHO images were used to measure the MVLL (Fig. 1) [8]. The morphological measurements of the anterior mitral leaflet (AML) and posterior mitral leaflet (PML) lengths were made using the apical three-chamber view (A3C) at end-diastole (*n* = 142, 92%), as well as coaptation length of the MVLs. Among patients with poor visualization in A3C, the measurements were conducted using the parasternal long-axis view (PLAX) (*n* = 9, 6%). Two ECHO examinations had poor visualizations and did not facilitate MV-specific measurements; hence, these MV-specific data were registered as missing (*n* = 2, 1%). Data from one patient with inaccessible ECHO images were registered as missing (*n* = 1, 1%). Elongated AML was considered at > 24 mm and elongated PML at > 14 mm [10].

### Invasive pressure measurements before, during, and after ASA

Invasive intracardiac pressure measurements were performed using the standard end-hole fluid-filled catheters, recorded, and stored using the Cardio Xper system (Philips Healthcare, Andover, MA, USA). The data from pressure measurements were collected continuously throughout the ASA: that is, the pressure gradients were measured invasively and simultaneously during CATH with one catheter placed in the LV and one in the ascending aorta. The following data were collected: heart rhythm and rate, LV peak systolic, early and late diastolic pressures, peak systolic and diastolic pressures in the ascending aorta, both at rest and during the Valsalva maneuver, and/or during induced premature ventricular contractions (PVCs). The peak-to-peak systolic pressure gradient across LVOT was calculated as the difference between LV and aortic peak systolic pressures. Resting and provoked LVOT peak-to-peak gradients were calculated before and 10 min after alcohol administration at the end of the ASA. The amount of injected alcohol was registered.

### Follow-up

The following data were collected from the 1-year follow-up: average days until follow-up, pacemaker (PM) or implantable cardioverter defibrillator (ICD) implantation, and the number of re-interventions (i.e., ASA or myectomy). ECHO data collected from the 1-year follow-up included: prevalence of SAM, BSWT, LV end-diastolic diameter, maximal LVOT velocity and gradients at rest and Valsalva maneuver, estimated from maximal LVOT velocity using a simplified Bernoulli equation (Δ*P* = 4*V*_max_^2^). The remaining LVOTO was defined as a peak instantaneous gradient across a LV outflow of ≥ 30 mmHg, either at rest or on provocation derived from the ECHO examination at the 1-year follow-up or from the most recent ECHO examination before reintervention for the patients who underwent reintervention.

### Statistical analysis

The Shapiro–Wilk test was used to verify whether the continuous data were normally distributed. The normally distributed data were presented with mean ± standard deviation (SD), while non-normally distributed data were presented with median and interquartile range (IQR). Binary variables were reported as absolute numbers and percentages. Logistic and linear regression models were used to evaluate the correlation between sex, AML and PML lengths, BSWT, amount of alcohol injected, and outcome (i.e., remaining LVOTO) after ASA. To evaluate the correlation between sex, the AML length, the PML length, the BSWT, the amount of alcohol injected, and the outcome (remaining LVOTO) after ASA. The linear regression models were adjusted for sex and age. Several cutoff values based on the different IQR values for BSWT were assessed using logistic regression to determine if any BSWT was associated with better outcomes at follow-up. The correlation between BSWT and remaining LVOTO at follow-up was evaluated by analyzing the area under the receiver operating curve (AUC). Inter- and intra-observer variability in the measures of BSWT, AML and PML lengths were calculated using intra-class correlation (ICC). *p* values < 0.05 were considered significant. The data analyses were conducted using STATA software (version 17.0; Stata Corp., College Station, TX, USA).

## Results

### Baseline characteristics and pre-procedural ECHO

The baseline characteristics from the pre-procedural ECHO are presented in Table 1. The study population consisted of 77 men and 77 women. The pre-procedural ECHO was conducted a maximum of 7 days before ASA, although 95% of the patients underwent the pre-procedural ECHO on the same day, or 1 day before ASA. The pre-procedural ECHOs showed that 147 (95%) patients had SAM (with or without septal contact) at rest. However, the patients without SAM at rest developed LVOTO with SAM at the Valsalva maneuver. The median BSWT before the procedure was 19 mm (IQR, 17–21 mm). The mean AML and PML lengths were 24.2 ± 3.2 mm and 16.3 ± 2.8 mm, respectively. Patients with pre-procedural SAM did not have significantly longer AML length (mean, 24.2 ± 3.2 mm) compared with patients without SAM (mean, 24.4 ± 2.6 mm, *p* = 0.827). In addition, patients with pre-procedural mitral regurgitation (grades 1–3, as no patient had grade 4 mitral regurgitation) did not have significantly longer AML length (*p* = 0.213) compared with no mitral regurgitation. The mean amount of alcohol injected during ASA was 1.6 ± 0.3 mL (IQR, 1.5–1.8 mL) while the mean amount per millimeter of BSWT was 0.85 ± 0.2 mL/mm.

**Table 1:** Patient characteristics and pre-procedural ECHO variables among patients with hypertrophic obstructive cardiomyopathy undergoing alcohol septal ablation

Patient characteristics	All patients (*n* = 154)	Women (*n* = 77)	Men (*n* = 77)	*p* value
Age mean ± SD, years	63 ± 12	64 ± 12	58 ± 11	**0.002**
Weight, median (IQR), kg	80 (70–95)	71 (61–85)	92 (78–103)	** < 0.001**
Height, mean ± SD, cm	170 ± 11	163 ± 7	178 ± 8	** < 0.001**
BSA, mean ± SD, m^2^	1.9 ± 0.3	1.8 ± 0.2	2.1 ± 0.2	** < 0.001**
Hypertension, *n* (%)	84 (54)	43 (56)	41 (53)	0.746
Coronary artery disease, *n* (%)	10 (6)	3 (4)	7 (9)	0.203
Hyperlipidemia, *n* (%)	6 (4)	1 (1)	5 (6)	0.133
Diabetes, *n* (%)	5 (3)	4 (5)	1 (1)	0.207
Renal failure, *n* (%)	4 (3)	2 (3)	2 (3)	1.00
COPD, *n* (%)	1 (1)	0 (0)	1 (1)	NA
Pre-procedural ECHO parameters				
LV end-diastolic diameter, median (IQR), mm	43 (39–46)	41 (37–45)	43 (40–47)	**0.007**
LV end-systolic diameter, mean ± SD, mm	27 ± 6	26 ± 6	27 ± 6	0.104
LV end-diastolic volume indexed BSA, median (IQR), mL	50 (38–58)	44 (35–57)	54 (48–62)	** < 0.001**
LV end-systolic volume indexed BSA, median (IQR), mL/m^2^	21 (14–26)	18 (13–24)	23 (17–27)	**0.021**
LA end-systolic volume indexed BSA, median (IQR), mL/m^2^	45 (33–54)	44 (31–54)	45 (38–54)	0.997
LV ejection fraction, mean ± SD, %	60 ± 8	60 ± 7	59 ± 9	0.268
Posterior wall diameter, median (IQR), mm	12 (10–14)	11 (9–13)	13 (10–15)	**0.027**
BSWT, median (IQR), mm	19 (17–21)	18 (16–20)	19 (17–21)	0.220
Mitral regurgitation, *n* (%)				
Grade < 1	55 (38)	29 (39)	26 (37)	0.614
Grade 1	69 (48)	32 (43)	37 (52)	0.418
Grade 2	20 (14)	13 (18)	7 (10)	0.156
Grade 3–4	1 (1)	0 (0)	1 (1)	NA
Aortic regurgitation, *n* (%)				
Grade < 1	125 (87)	61 (83)	64 (90)	0.417
Grade 1	16 (11)	10 (14)	6 (8)	0.296
Grade 2	3 (2)	2 (3)	1 (1)	0.567
Grade 3–4	0 (0)	0 (0)	0 (0)	NA
SAM, *n* (%)	147 (95)	74 (96)	73 (95)	0.700
LVOTO of ≥ 30 mmHg at rest or at Valsalva maneuver, *n* (%)	154 (100)	77 (100)	77 (100)	NA
LVOT gradient at Valsalva maneuver, mean ± SD, mmHg	97.5 ± 58.5	108.7 ± 61.4	86.0 ± 53.0	0.018
AML, mean ± SD, mm	24.2 ± 3.2	24.3 ± 2.8	24.1 ± 3.5	0.729
AML-indexed BSA, mean ± SD, mm/m^2^	12.7 ± 2.4	13.7 ± 2.1	11.6 ± 2.3	** < 0.001**
PML, mean ± SD, mm	16.3 ± 2.8	15.6 ± 1.9	16.9 ± 3.4	**0.007**
PML-indexed BSA, mean ± SD, mm/m^2^	8.5 ± 1.8	8.8 ± 1.5	8.1 ± 1.9	**0.018**
Coaptation length of the mitral valve leaflets, mean ± SD, mm	8.8 ± 1.8	8.4 ± 1.7	9.2 ± 1.8	**0.007**

The table presents the patient characteristics and results as absolute numbers (*n*) and percentages (%)

*AML* anterior mitral valve leaflet, *BSA* body surface area, *BSWT* basal septal wall thickness, *COPD* chronic obstructive pulmonary disease, *IQR* interquartile range, *LVOT* left ventricular outflow tract, *LVOTO* left ventricular outflow tract obstruction, *NA* not applicable, *PML* posterior mitral valve leaflet, *SD* standard deviation, *SAM* systolic anterior motion

*p*-values present comparisons (linear or logistic regression models) between women and men, while significant *p* values are shown in bold type

Normally distributed data are presented as mean ± SD while non-normally distributed data are presented as medians with IQR

### Intra-procedural invasive measurements and acute hemodynamic effects of ASA

**Fig. 2:**
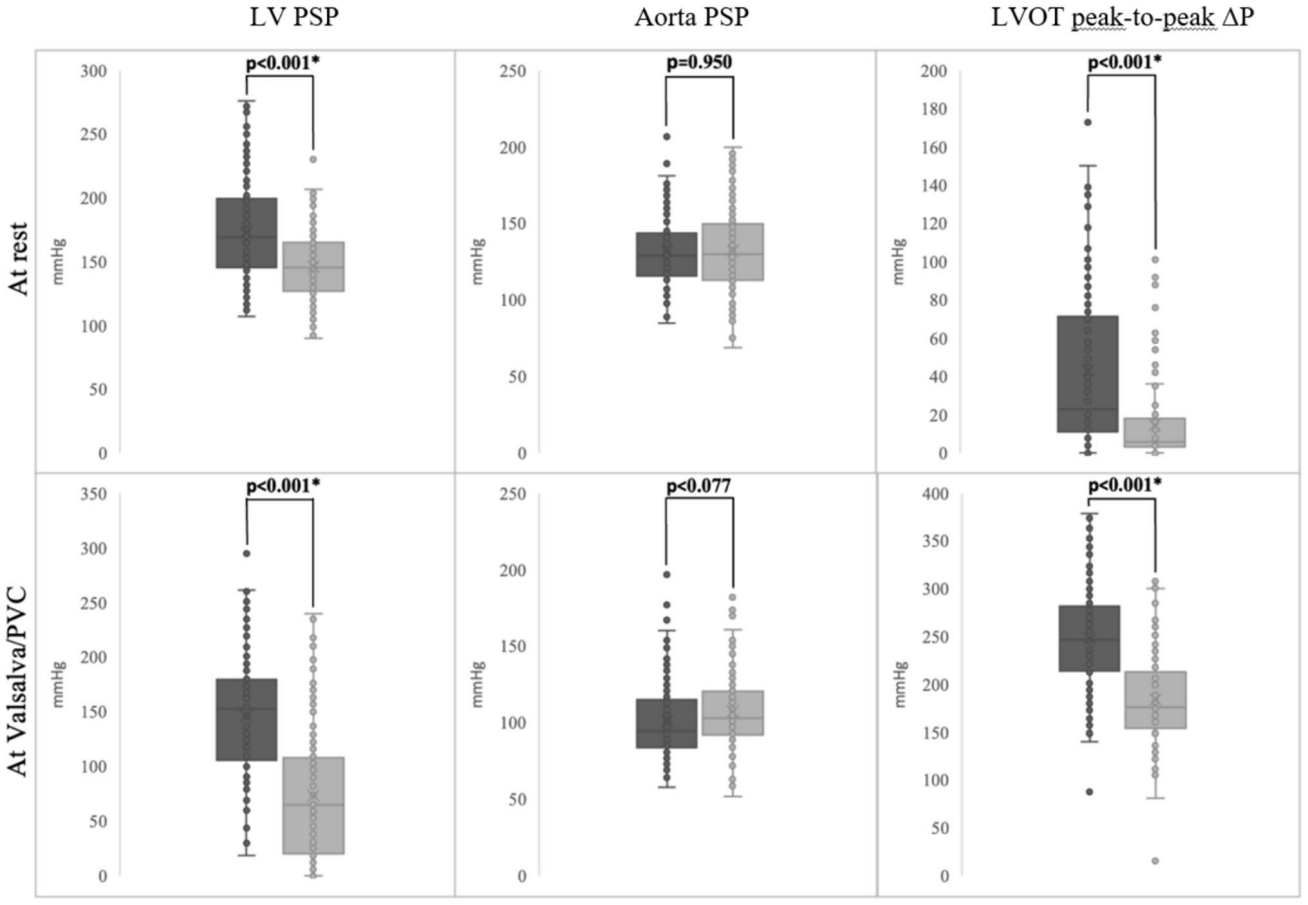
Comparison between invasive systolic left ventricular pressure gradients before (dark gray) and after (light gray) alcohol septal ablation at rest and during Valsalva maneuver/PVC. Values are expressed as median and interquartile ranges. *Statistically significant *p* values. *LVOT* left ventricular outflow tract, *PSP* peak systolic pressure, *PVC* induced premature ventricular contraction

The systolic pressures and peak-to-peak systolic pressure gradients pre- and post-ASA at rest and at Valsalva maneuver/PVC are presented in Fig. 2. There was no significant change in the systolic aortic pressure pre- and post-ASA; however, there was a significant decrease in the LV peak systolic pressure and LVOT peak-to-peak pressure gradient post-ASA compared to pre-ASA, at both rest and Valsalva maneuver. Patients with elongated AML did not have higher pre-procedural LV peak systolic pressure either at rest (173.0 ± 37.1 mmHg versus 173.5 ± 39.9 mmHg, *p* = 0.940) or at Valsalva maneuver/PVC (148.5 ± 55.3 mmHg versus 144.9 ± 55.2 mmHg, *p* = 0.708), compared with patients with AML < 24 mm. No difference in post-procedural LV peak systolic pressure was seen in patients with elongated AML either at rest (*p* = 0.150) or at Valsalva maneuver/PVC (*p* = 0.915) compared with patients with AML < 24 mm.

### Clinical outcomes and follow-up echocardiography

The follow-up ECHO was performed with a median of 364 days post-procedure (IQR 334–385 days). During this follow-up period, 13 (8%) patients underwent a new ASA, and 1 (1%) patient required post-procedural myectomy. The patients who underwent reintervention had the most recent ECHO examination before reintervention registered at their follow-up ECHO. Between the ASA and the follow-up, 34 patients were implanted with either permanent PM (*n* = 29, 19%) or ICD (*n* = 5, 3%). There was no significant difference in LVOT gradient either at rest (*p* = 0.089) or at Valsalva maneuver (*p* = 0.067) at the 1-year follow-up among the patients who received a permanent PM after ASA (rest: 14.7 ± 9.3 mmHg, Valsalva maneuver: 33.2 ± 31.4 mmHg) and those who did not require a PM (rest: 22.0 ± 22.4 mmHg, Valsalva maneuver: 49.3 ± 44.2 mmHg).

The results from the follow-up ECHO examinations showed that 85 (57%) patients had remaining SAM (with or without septal contact) at follow-up; however, 128 (86%) patients no longer had LVOTO after ASA. Sixty-three patients (42%) had some degree of mitral regurgitation at follow-up (Appendix 1). The amount of alcohol injected during the procedure was not associated with remaining LVOTO at follow-up (*p* = 0.865). The patients with remaining LVOTO or SAM at follow-up were not significantly older (*p* = 0.096 and *p* = 0.282, respectively).

There was a significant reduction in septal thickness as shown by the pre-procedural and follow-up ECHOs (pre-procedural, 19 mm [IQR, 17–21 mm]; follow-up, 16 mm [IQR, 13–18 mm]; *p* < 0.001). Among all patients, 19 (12%) had BSWT ≥ 23 mm, which was significantly associated with remaining LVOTO at follow-up, while 7 (37%) patients with BSWT ≥ 23 mm had remaining LVOTO at follow-up, compared to 11% of the patients with BSWT < 23 mm, (AUC for BSWT cutoff ≥ 23 mm = 0.73; *p* = 0.004) (Table 2). There was no difference in post-procedural CATH gradients or the prevalence of remaining SAM or mitral regurgitation at the 1-year follow-up compared with the patients with BSWT ≥ 23 mm and those with BSWT < 23 mm (Appendix 1).

**Table 2:** Basal septal wall thickness, anterior and posterior mitral leaflet length, and amount alcohol in relation to remaining left ventricular outflow tract obstruction and reablation after alcohol septal ablation

	No LVOTO at follow-up, *n* = 128	Remaining LVOTO, *n* = 21	*p* value*	Cutoff (mm)	*p* value**
BSWT, median (IQR)	19.0 (17–21)	20.2 (18–24)	**0.039**	> 23	**0.004**
AML, mean ± SD, mm	24.1 ± 3.2	24.5 ± 3.3	0.592	> 24	0.269
PML, mean ± SD, mm	16.3 ± 2.9	16.1 ± 2.4	0.730	> 14	0.472
Alcohol injected, mean ± SD, mL	1.64 ± 0.3	1.62 ± 0.4	0.734	–	–
Alcohol per 10 mm BSWT, mean ± SD, mL/ 10 mm	0.86 ± 0.2	0.82 ± 0.3	0.494	–	–

*p values obtained from linear regression models adjusted for sex and age

**p values obtained from logistic regression models with groups arranged by cutoff, adjusted for sex and age. Statistically significant *p* values are shown in bold type. Normally distributed data are presented as mean ± SD while non-normally distributed data are presented as medians with IQR

*AML* anterior mitral leaflet, *BSWT* basal septal wall thickness, *IQR* interquartile range, *LVOTO* left ventricular outflow tract obstruction, *PML* posterior mitral valve leaflet

Elongated MVLL (either AML or PML) was present in 125 (90%) patients. Elongated AML (> 24 mm) was present in 67 (44%) patients, although AML length was not associated with remaining LVOTO at follow-up (Table 2). AML was not associated with post-procedural SAM (*p* = 0.342) or mitral regurgitation (*p* = 0.378). Elongated PML (> 14 mm) was present in 114 (74%) patients, but PML was not associated with remaining LVOTO at follow-up (Table 2).

### Sex differences

There were no significant differences in outcomes between men and women when comparing CATH or ECHO parameters (Table 3). There was no difference in remaining LVOTO between women and men at post-procedural CATH or follow-up (Table 3). Men had significantly longer PML compared with women, although both AML and PML lengths indexed for BSA were longer among women (Table 1).

**Table 3:** Sex differences at 1-year follow-up after alcohol septal ablation in patients with hypertrophic obstructive cardiomyopathy

Clinical outcomes	All patients	Men (*n* = 77)	Women (*n* = 77)	*p* values
Myectomy, *n* (%)	1 (1)	0 (0)	1 (1)	
Reablation, *n* (%)	13 (9)	5 (7)	8 (11)	0.375
Pacemaker, *n* (%)	29 (20)	14 (18)	15 (20)	0.806
ICD, *n* (%)	5 (3)	2 (3)	3 (4)	0.641
Post-procedural CATH outcomes, *n* = 154				
Remaining LVOTO at rest, *n* (%)	29 (19)	11 (14)	18 (23)	0.153
At least 50% reduction of acute LVOT gradient at rest, *n* (%)	68 (44)	30 (40)	38 (51)	0.165
Remaining LVOTO at Valsalva maneuver/PVC, *n* (%)	153 (99)	76 (99)	77 (100)	
At least 50% reduction of acute LVOT gradient at Valsalva maneuver/PVC, *n* (%)	83 (54)	40 (56)	43 (60)	0.682
Follow-up ECHO outcomes, *n* = 149				
Remaining LVOTO, *n* (%)	21 (14)	8 (11)	13 (18)	0.230
At least 50% reduction of LVOT gradient at rest, *n* (%)	72 (48)	32 (43)	40 (54)	0.165
At least 50% reduction of LVOT gradient at Valsalva maneuver, *n* (%)	82 (55)	40 (54)	42 (58)	0.602
Base septal thickness reduction, mean ± SD, mm	– 3.4 ± 4.1	– 3.3 ± 3.9	– 3.5 ± 4.1	0.700

Values are expressed as absolute numbers (*n*) and percentages (%) or mean ± standard deviation for normally distributed variables. *p* values were obtained from logistic regression models. *p* value was left out for myectomy as no men underwent myectomy

*ASA* alcohol septal ablation, *ECHO* echocardiography, *ICD* implantable cardioverter defibrillator, *CATH* invasive intracardiac pressure examination, *LVOT* left ventricular outflow tract, *PVC* induced premature ventricular contraction, *SD* standard deviation

### Peri- and post-procedural complications

In total, 30 (19%) patients received either atrioventricular (AV) blocks II or III between the ASA and the 1-year follow-up. AV blocks II or III were more common among women (*n* = 20, 26%) compared with men (*n* = 10, 13%, *p* = 0.045). Four patients (3%) received atrial fibrillation after ASA, one patient (1%) had post-procedural tamponade, and one (1%) had cardiac arrest. One patient died within 30 days after the ASA, while three patients died within 1 year after ASA. There were no sex differences in mortality (*p* = 0.225).

### Intra- and inter-observer analysis

The intra- and inter-observer agreements for BSWT and MVLL measurements were analyzed (Table 4).

**Table 4:** Intra- and inter-observer agreement

	Inter-observer	Intra-observer
	ICC	ICC
BSWT	0.92, *p* = 0.001	0.89, *p* = 0.001
AML	0.65, *p* = 0.035	0.83, *p* = 0.004
PML	0.89, *p* = 0.001	0.93, *p* < 0.001

Intra-class correlation coefficients (ICCs) were obtained using two-way mixed-effects models as a measure of agreement

*AML* anterior mitral valve leaflet, *BSWT* basal septal wall thickness, *ICC* intra-class correlation coefficients, *PML* posterior mitral valve leaflet

## Discussion

This single-center retrospective cohort study assessed whether there were any associations between BSWT, AML and PML lengths, sex, and outcomes in patients who received ASA treatment for HOCM. In general, patients with BSWT ≥ 23 mm had a significantly higher rate of remaining LVOTO at follow-up. Most patients had elongated MVLL; however, AML and PML lengths were not associated with remaining LVOTO at follow-up. In addition, the amount of alcohol injected during ASA was not associated with remaining LVOTO at follow-up. These results suggest that BSWT could be a possible predictive value for LVOTO reduction after ASA; however, further studies of the potential anatomical predictors for patients with HOCM to optimize treatments based on individual conditions are required.

### Basal septal thickness and remaining LVOTO

At the 1-year follow-up, 14% of patients had remaining LVOTO, which is supported by a recent ASA review showing that 10%-20% of patients have a residual gradient > 30 mmHg after ASA [11]. In addition, considering that BSWT ≥ 23 mm had a significantly higher rate of remaining LVOTO at follow-up, it has previously been shown that BSWT is associated with LVOTO in patients with HOCM [11]. Jensen et al*.*’s study of 531 patients with HOCM using a follow-up time of 0.6 ± 0.6 years after ASA showed that patients with a thicker septum had a higher residual LVOTO on follow-up compared to patients with a thinner septum [12]. Their study used three cutoffs (i.e., < 20 mm, 20–24 mm, and ≥ 25 mm), where the ≥ 25 mm group showed a trend of higher residual gradients on follow-up, although this was not significant when compared with the other groups [12]. A study of 102 patients with HOCM by Lu et al. found that a septum thinner than 24.3 mm had a higher rate of LVOT gradient reduction, which was defined as a 50% reduction compared to the gradient before ASA, while patients with a septal thickness of ≥ 24.3 mm had more non-responders [13]. Lulu et al*.* suggested using 24.3 mm as a cutoff when considering treating patients with ASA because a significant number of patients with a septum thicker than 24.3 mm were non-responders and had worse outcomes in LVOT gradient reduction [13]. In contrast to our study, however, Lu et al*.* used a different definition for LVOT reduction, with a 50% reduction in LVOT gradient between pre-procedural examination and follow-up. In summary, BSWT seems to be a promising predictive value for LVOTO reduction after ASA. Given a non-prohibited surgical risk, septal myectomy could be a better suitable method for the treatment of severe LVOTO in patients with a very thick septum; however, this must be studied further.

In this study, the amount of alcohol injected during the procedure was not associated with remaining LVOTO at follow-up. The mean amount of alcohol injected during the ASA treatment in this study (1.6 ± 0.3 mL) was lower than in previous studies reporting mean alcohol doses of 2.1–2.2 mL [14]. However, the BSWT in this study was lower (mean, 19 mm) than in earlier studies (mean, 20.1–20.7 mm). Although the recommended dose of alcohol in ASA is 1 mL per 10 mm of BSWT [15], the amount of alcohol per 10 mm of BSWT in this study equaled 0.85 ± 0.2 mL/mm, which was lower than the recommended dose. This could explain some of the cases of remaining LVOTO after ASA. The reasons for using a lower dose of alcohol than 1 mL per 10 mm of BSWT included contrast uptake (indicating possible alcohol exposure) in the right ventricular (RV) myocardium, contrast bubbles in LV or RV lumen directly after alcohol injection, indicating possible endocardial alcohol exposure, or difficulty injecting alcohol into the septal branch due to catheter blockage after partial alcohol injection. At the 1-year follow-up, one patient had undergone surgical myectomy, and 13 (9%) patients had undergone reablation. Therefore, seven patients with remaining LVOTO after ASA did not undergo reintervention during the first year after ASA. However, some patients could have undergone reintervention later than 1 year after ASA or their symptoms might have been relieved, as the LVOT gradient could have been reduced.

### Mitral valve impact in patients with HOCM

Elongation of the AML and PML (compared to normal reference range [10]) was observed in most patients in this study, in line with previous studies of patients with HOCM suggesting elongated MVLs as being part of the innate HCM phenotype [10, 16–18]. Moreover, we found that BSA-indexed MVLL was significantly longer in women, highlighting a possible persistent functional difference that must be studied further. The pathophysiology of SAM in HOCM comprises an interaction between the hypertrophic morphology, abnormalities in the MV anatomy, and labile hemodynamic derangements, which cause drag forces that are responsible for SAM [2]. As MV abnormalities contribute to LVOT obstruction, septal myectomy may be combined with concomitant MV surgery to reduce SAM or SAM-mediated mitral regurgitation [19]. Several methods have been described, such as AML extension using bovine pericardium, stiffening the leaflet, and enhancing coaptation posteriorly, the “resect-plicate-release” procedure, AML extension, surgical edge-to-edge MV repair, AML retention plasty, or secondary chordal cutting [2, 19]. Septal myectomy with concomitant MV surgery has been shown to be equally safe and successful as isolated myectomy for the treatment of HOCM [20]. Hence, concomitant MV surgery is a possible advantage for surgical approaches during ASA. Freedom from reintervention as well as early and late reduction of LVOT gradient are superior in patients undergoing septal myectomy, although survival is equal in patients undergoing myectomy and ASA [21]. To our knowledge, the correlation between MVLL and outcome after ASA has not yet been shown. In our study, MVLL was not associated with remaining LVOTO at follow-up. AML length was not associated with SAM or mitral regurgitation (at both pre-procedural and follow-up), while patients with elongated AML (> 24 mm) did not have higher invasively measured peak systolic LVOT gradient (either pre- or post-procedural) than patients with AML < 24 mm. This could indicate that the patients in our study would maybe not have benefited from concomitant MV surgery, although this conclusion does not include other possible MV abnormalities. However, interventional treatment options for patients with symptomatic HOCM, with or without mitral regurgitation and/or MVL elongation, should be evaluated carefully to enable the best outcomes for individual patients.

### Sex differences in patients with HOCM undergoing ASA

There was no difference in the prevalence of remaining LVOTO at follow-up between women and men; however, the LVOT gradient at the pre-procedural ECHO was significantly higher among women. In addition, women were significantly older at the time of ASA and had a higher prevalence of postoperative complications (i.e., AV blocks) compared with men. This finding was supported by the results of a study by Saravanabavanandan et al*., which* showed that women were older at the time of intervention, had higher short-term all-cause mortality, as well as a higher incidence of atrioventricular block, permanent PM implantation, and hospital stay after ASA or myectomy, compared to men [22]. These findings suggest that women might be experiencing a more severe disease stage than men at the time of intervention, where they may have a higher risk for postoperative complications due to their late age and advanced disease. Nevertheless, this must be studied further.

### Strengths and limitations

This study was performed as a retrospective cohort study among patients diagnosed with HOCM undergoing ASA. One of the strengths of this study was the amount of data collected and reported for all included patients, which facilitated an evaluation with few excluded data points. In addition, the study population had an equal gender distribution, and the number of patients (*n* = 154) was satisfactory. The study-specific variables (i.e., AML and PML lengths) were measured by two observers according to a previously described methodology and were discussed with the team when necessary. The measurement methodology corresponded with previous studies [7, 9, 17]. A potential limitation could be that the examinations were performed by different examiners which may affect the repeatability of the results. In this study, two patients (equal to 1%) were excluded from the MV-specific measurements due to inadequate ECHO quality. In the pre- and post-procedural examinations, a total of 14 patients (9%) were measured in PLAX view instead of A3C view due to poor visualization, which could have only a limited impact on the results. The ICC values indicated good reliability for BSWT and PML length measurements; however, they showed a more moderate reliability for AML length. AML length can sometimes be problematic to measure, as it can be difficult to identify the transition between leaflet and chordae.

We have also shown that patients with a basal interventricular septum ≥ 23 mm had a lesser reduction of LVOTO after ASA; therefore, they may need a longer follow-up for reevaluation.

## Conclusion

This study suggests that pre-procedural septal thickness ≥ 23 mm was associated with remaining LVOTO at follow-up 1 year after ASA. AML and PML lengths and sex do not seem to contribute significantly to remaining LVOTO. Most patients with HOCM had elongated MVLL, which supports the notion of elongated MVLs in patients with HOCM. In summary, these findings suggest that BSWT measured with ECHO can be used effectively in patient selection for successful ASA. A severe basal septal hypertrophy in patients with HOCM may be associated with a poor outcome after ASA. Further studies of potential sex differences and detectable differences in the cardiac anatomy of patients with HOCM are necessary to optimize treatments based on individual conditions.

## Data Availability

The data that support the findings of this study are available from the corresponding author, upon reasonable request.

## Appendix 1. Basal septal wall thickness and its relation to outcomes at follow-up after alcohol septal ablation

**Table:**

Clinical outcomes	All patients	BSWT < 23 mm (*n* = 135)	BSWT ≥ 23 mm (*n* = 19)	*p* value
Myectomy, *n* (%)	1 (1)	1 (1)	0 (0)	NA
Reablation, *n* (%)	13 (9)	12 (9)	1 (5)	0.599
Pacemaker, *n* (%)	29 (20)	27 (20)	2 (11)	0.333
ICD, *n* (%)	5 (3)	3 (2)	2 (11)	0.083
Post-procedural CATH outcomes, *n* = 154				
Remaining LVOTO at rest, *n* (%)	29 (19)	24 (18)	5 (26)	0.377
Remaining LVOTO at Valsalva maneuver/PVC, *n* (%)	153 (99)	134 (99)	19 (100)	NA
1-year follow-up ECHO outcomes, *n* = 149				
LVOTO, *n* (%)	21 (14)	14 (10)	7 (37)	**0.023**
SAM, *n* (%)	85 (57)	72 (53)	13 (68)	0.273
Mitral regurgitation, *n* (%)	63 (42)	53 (61)	10 (63)	0.331
Grade 1	54 (36)	45 (34)	9 (47)	
Grade 2	8 (5)	7 (5)	1 (5)	
Grade 3	1 (1)	1 (1)	0 (0)	

Values are expressed as absolute numbers (*n*) and percentages (%). *p* values were obtained from logistic regression models. None of the patients had grade 4 mitral regurgitation

*ASA* alcohol septal ablation, *ECHO* echocardiography, *ICD* implantable cardioverter defibrillator, *CATH* invasive intracardiac pressure examination, *NA* not applicable, *PVC* induced premature ventricular contraction
